# The Potential of Human Peptide LL-37 as an Antimicrobial and Anti-Biofilm Agent

**DOI:** 10.3390/antibiotics10060650

**Published:** 2021-05-29

**Authors:** Kylen E. Ridyard, Joerg Overhage

**Affiliations:** Department of Health Sciences, Carleton University, Ottawa, ON K1S 5B6, Canada; kylenridyard@cmail.carleton.ca

**Keywords:** LL-37, antimicrobial peptide, anti-biofilm peptide, antimicrobial resistance, LL-37 derivatives

## Abstract

The rise in antimicrobial resistant bacteria threatens the current methods utilized to treat bacterial infections. The development of novel therapeutic agents is crucial in avoiding a post-antibiotic era and the associated deaths from antibiotic resistant pathogens. The human antimicrobial peptide LL-37 has been considered as a potential alternative to conventional antibiotics as it displays broad spectrum antibacterial and anti-biofilm activities as well as immunomodulatory functions. While LL-37 has shown promising results, it has yet to receive regulatory approval as a peptide antibiotic. Despite the strong antimicrobial properties, LL-37 has several limitations including high cost, lower activity in physiological environments, susceptibility to proteolytic degradation, and high toxicity to human cells. This review will discuss the challenges associated with making LL-37 into a viable antibiotic treatment option, with a focus on antimicrobial resistance and cross-resistance as well as adaptive responses to sub-inhibitory concentrations of the peptide. The possible methods to overcome these challenges, including immobilization techniques, LL-37 delivery systems, the development of LL-37 derivatives, and synergistic combinations will also be considered. Herein, we describe how combination therapy and structural modifications to the sequence, helicity, hydrophobicity, charge, and configuration of LL-37 could optimize the antimicrobial and anti-biofilm activities of LL-37 for future clinical use.

## 1. Introduction

A recent report by the Interagency Coordination Group on Antimicrobial Resistance (IACG) indicated that drug-resistant diseases are the cause for over 700,000 deaths per year worldwide. Without proper intervention, this value is projected to increase to 10 million deaths annually by the year 2050. As such, antibiotic resistance is now considered to be one of the biggest threats to global health [1]. As antibiotics are becoming increasingly ineffective, it is becoming more difficult to treat common infectious diseases, such as tuberculosis and pneumonia. To avoid a post-antibiotic era, it is imperative that new antimicrobial agents and strategies are developed [2]. Since antimicrobial peptides (AMPs), also known as host defense peptides, were discovered in the 1980s, they have been viewed as a promising alternative to conventional antibiotics [3].

In general, AMPs are cationic, amphipathic, alpha helical molecules consisting of 12–100 amino acids [4]. These antimicrobial agents primarily kill bacteria via membrane interactions [5]. This mechanism of action differs from that of traditional antibiotics; antibiotics are generally bacteriostatic and target specific cellular processes, whereas AMPs are bactericidal with multiple potential targets aside from its characteristic membrane disruptions [6]. For this reason, AMPs have been considered to be less prone to resistance than conventional antibiotics. AMPs are regarded as a potential therapeutic agent for their broad-spectrum activities and immunomodulation, as well as their direct killing abilities and low resistance rates [7].

There are two subfamilies of AMPs in mammals: Cathelicidins and defensins. Both types of AMPs are part of the innate immune system. In humans, there are many classes of defensins, while there is only a single identified cathelicidin [5]. This review focuses on the antimicrobial effects of the human cathelicidin, LL-37, and its potential as a novel peptide antibiotic.

The precursor to LL-37, hCAP-18, is stored in cytoplasmic granules and lamellar bodies [8,9]. The inactive form of LL-37 resides in the C-terminal domain of hCAP-18 and must be proteolytically activated by enzymes kallikreins or proteinase 3. Once cleaved, LL-37 is further processed to a 37 amino acid peptide [9]. The synthesis and secretion of LL-37 is seen mostly in epithelial cells, although immune cells including macrophages, dendritic cells, natural killer cells, neutrophils, and mesenchymal stem cells have also been shown to secrete LL-37. The expression of LL-37 in most epithelia is constitutive and is regulated by cAMP-signaling pathways, though the expression of LL-37 can also be induced in keratinocytes [8]. Many factors, such as pathogen-associated molecular patterns and pro-inflammatory cytokines, can induce the expression of LL-37, allowing for a high concentration to accumulate at the site of infection [10].

LL-37 has moderate antimicrobial activities against numerous Gram-negative and Gram-positive bacteria, including pathogens from the *Pseudomonas*, *Escherichia*, *Staphylococcus*, and *Enterococcus* genera [11]. LL-37 is able to kill bacteria through direct antibacterial activities, as well as through immunomodulation. Like other AMPs, the primary mechanism of action is membrane disruption. The net positive charge of +6 allows for LL-37 to bind to the negatively charged membrane of bacteria. Upon binding, the introduction of transmembrane pores causes a disruption of cell integrity that leads to cell lysis and death. In addition, LL-37 is able to permeate the cell membrane in order to interact with intracellular targets, such as acyl carrier proteins [12,13]. LL-37 has additional immunomodulatory properties, including both pro-inflammatory and anti-inflammatory responses, that are important for the indirect killing of bacteria. For example, LL-37 is capable of inducing cell migration, proliferation and differentiation. While the immunomodulatory activities of LL-37 play an important role in the killing of bacteria, it is not the focus of the review as these roles have been previously covered in several other analyses [14,15,16,17].

Despite the promising antibacterial and immunomodulatory activities, LL-37 and its derivatives have yet to achieve regulatory approval as a therapeutic agent [3]. In this review, we will discuss the existing limitations of LL-37 as an antimicrobial agent, with an emphasis on the mechanisms of bacterial resistance. Then, we will summarize the strategies that can optimize LL-37’s potential as a novel peptide antibiotic.

## 2. Challenges Associated with Using LL-37 as an Antimicrobial Agent

### 2.1. Resistance

Previous findings suggest that the likelihood of AMP resistance is low in comparison to antibiotic resistance, yet a review by Maria-Neto et al. described the development of AMP resistance as inevitable [18]. Several studies have examined the ability of bacteria to acquire resistance following serial passages of sub-lethal concentrations of LL-37 [19,20,21,22]. The use of sub-minimal inhibitory concentration (MIC) values of LL-37 led *Staphylococcus aureus* to develop resistance after three passages [22], and was found to develop stable resistance within 168 generations of increasingly large concentrations of LL-37 [19]. Multiple more studies have reported the induction of AMP-resistant phenotypes following prolonged exposure to LL-37, including in *Salmonella typhimurium* [23] and *Clostridioides difficile* [21].

**Figure 1:**
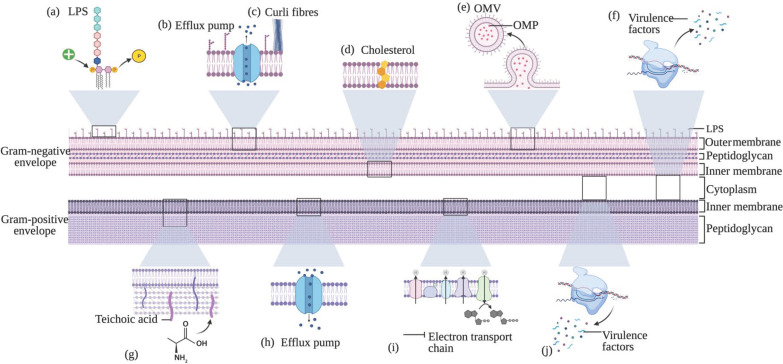
Schematic presenting examples of major cellular resistance mechanisms seen in Gram-negative and Gram-positive bacteria that work to decrease its susceptibility to LL-37. Mechanisms (**a**–**f**) are specific to the Gram-negative bacterial envelope. (**a**) LPS modifications as an example of cell membrane and charge modifications that occur in the outer membrane, (**b**) efflux pumps, (**c**) curli production, (**d**) cholesterol as an example of the incorporation of exogenous molecules into the membrane, (**e**) the formation of outer membrane vesicles (OMVs) with outer membrane proteins (OMPs) as an example of a component that may be transported via OMVs, and (**f**) the upregulation of virulence factors that occur in the cytoplasm. Mechanisms (**g**–**j**) are specific to the Gram-positive envelope. (**g**) D-alanination of teichoic acid as an example of cell membrane and charge modifications that occur in the inner membrane, (**h**) efflux pumps, (**i**) inhibition of the electron chain (ETC) as an example of a metabolic change in the cell, and (**j**) the upregulation of virulence factor expression that occur in the cytoplasm.

AMPs have evolved alongside bacteria over millions of years, and thus bacteria have acquired several mechanisms of resistance against LL-37 [24]. A summary of the resistance mechanisms can be seen in Figure 1. Most commonly, resistance to LL-37 occurs as a result of structural changes to the bacterial membranes [7], including modifications to the cell membrane [20], cell surface charge [9,21], capsule [25], and efflux pumps [26]. Furthermore, resistance may develop through the upregulation and downregulation of specific genes [25,27], and through the resulting alterations to cellular processes and secretions, including metabolism [19], the expression of virulence factors [28], proteases [12,27,29], and outer membrane proteins (OMP) and vesicles [12,27,29]. In this review, the mechanisms of resistance seen in Gram-positive and Gram-negative bacteria will be described.

#### 2.1.1. Cell Membrane and Charge Modifications

To evade LL-37, bacteria can alter their membranes in two ways: Modification to the structural composition [30] and/or by increasing the net charge [31,32,33]. For instance, in multi-drug resistant enterococci, the LiaFSR stress response system triggers phospholipid redistribution to avert antimicrobial agents away from its septal targets [30]. Multiple bacteria have also been reported to induce charge alterations to the outer surface or the cell membrane, effectively reducing the ability of antimicrobials to bind and penetrate the bacterial defenses [31,32]. In addition, increasing the cell surface charge in *S. aureus* was found to inhibit the bactericidal effects of basic AMPs [33]. The modifications seen in Gram-positive and Gram-negative bacteria are different considering their dissimilarities in the structural makeup of the membranes, and thus we will describe the changes separately. Overall, evidence suggests that a potential method to decrease resistance to LL-37 is through interfering with the bacterial cell wall synthesis and the genes responsible for its cell wall maintenance [34].

In Gram-positive bacteria, there are multiple enzymes responsible for incorporating positively charged molecules into the membrane, including flippase Tacf, LicD1, and sortase SrtA [35]. A common method to increase tolerance of Gram-positive bacteria to cationic AMPs (CAMPs) is through the addition of D-alanine and L-lysine molecules to the teichoic acid and the phosphatidylglycerol structures, respectively [21,36]. The protonated D-alanyl residues are positively charged and thus act to lower LL-37 binding efficacy [32]. The *dlt* operon is responsible for alanine incorporation, and it is common to nearly all Gram-positive bacteria, including *S. aureus*, *S. mutans*, *Streptococcus pyogenes*, *Streptococcus agalactiae*, *Streptococcus gordonii* [37], *Streptococcus suis* and *Streptococcus pneumoniae* [32]. Moreover, the addition of L-lysine residues to the phosphatidylglycerol leads to an increased tolerance to LL-37, and is regulated by the *mprF* gene [32,37,38]. The ApsTC two-component system (TCS) [37] and the GraRSX-VraGF five-component system [33] are known to regulate both the *dlt* operon and the *mprF* genes. It was reported in multiple studies that mutations within the *dlt* operon increases the susceptibility of bacteria to LL-37 [12,38]. Mutations within this gene have been shown to reduce the antimicrobial tolerance of *S. aureus* [38], yet the deletion in *S. agalactiae* did not alter the vulnerability to LL-37 [32], and the effect of lysinylation in *C. difficile* is unknown [21]. An example of a membrane modification that is not associated with charge alterations is a mutation in the Lancefield group A carbohydrate of *S. pyogenes*, specifically in the *N*-acetylglucosamine (GlcNAc) side-chain. Mutations of the *gacI* glycosyltransferase, which is essential for GlcNAc expression, leads to a higher sensitivity of *S. pyogenes* to LL-37 [39].

To increase the resistance to LL-37, Gram-negative bacteria can modify the LPS in order to enhance their abilities to evade bacteria. For instance, lipid A modifications in *Helicobacter pylori* alters O-antigen expression [40]. The LPS modifications frequently decrease the affinity of LL-37 to the outer bacterial membrane [25,26,27,40], typically through the addition of positively charged substituents, or the removal of phosphate groups from the lipid A disaccharide backbone [40]. When bacteria detect the presence of antimicrobials, the PhoPQ system induces three structural changes to the lipid A sequence: The addition of a palmitoyl chain [26], a hydroxyl group [26], or an aminoarabinose residue [26,41]. Another lipid A modification includes the increase of glucosamine [42]. The RamA global transcription regulator binds and activates three genes that are correlated with lipid A biosynthesis: *lpxC*, *lpxL-2* and *lpxO*. The activation induced by RamA has been shown to protect *Klebsiella pneumoniae* from degradation from AMPs [43]. Likewise, Cullen et al. found that the *lpxE* and *lpxF* genes in *H. pylori* are responsible for removing phosphate groups for the lipid A, thus promoting resistance and colonization. When the *lpxE* gene is deleted, the bacteria confer partial resistance, suggesting that the l*pxF* has the greatest impact on bacterial tolerance to host immune peptides [40]. The *waa* gene cluster also has a role in LPS biosynthesis and modification [23]. Within this cluster, *waaY* is responsible for adding a phosphate group to LPS; mutations in this gene increase the charge of the outer surface and therefore contributes to a lower vulnerability to LL-37 [23,44]. In fact, when *S. enterica* Typhimurium is under selective pressure from LL-37, it has been shown to induce mutations in the *waaY* gene in order to promote this resistant phenotype [23]. In contrast, mutations in the *waaP* gene lead to the hypersensitive deep-rough phenotype because, unlike the *waaY* mutation, mutations in the *waaP* gene leads to instability of the LPS [44]. Other modifications that led to a more positive charge, such as the addition of phosphoethanolamine (PEA) into the LPS, did not confer a higher tolerance to LL-37 [45].

#### 2.1.2. Efflux Pumps

Efflux pumps are a common resistance mechanism in both the cytoplasmic membranes of Gram-positive and Gram-negative pathogens. A review by Webber and Piddock suggests that the intrinsic antibiotic resistance of some bacterial species may be primarily due to the presence of efflux pumps [46]. In general, efflux pumps increase virulence through the ejection of AMPs from within the cells to the external environment, thus preventing LL-37 from exerting its antibacterial effects [27]. The upregulation of efflux pumps has been shown to enhance resistance to LL-37 and other antimicrobial agents [9,47], yet the upregulation may also serve as a disadvantage as nutrients and metabolic intermediates may be extruded along with the toxic substrates [19,46].

One specific mechanism of resistance is a detoxification module, consisting of a TCS and an efflux pump belonging to the ATP-binding cassette (ABC) superfamily [48,49,50]. When the presence of LL-37 is detected, the TCS will promote the expression of the transporter, thus resulting in the extrusion of LL-37 [48]. Examples of the detoxification modules include YxdJK-LM in *Bacillus subtilis* [48], and *graRS*-*vraFG* and *braRSAB* in *S. aureus* [49]. Several bceR-like systems in Gram-positive bacteria have been reported to include AMP detoxifications molecules, with one notable example being the bacitracin resistance module (*bceRSAB*) of *B. subtilis* [49,50]. Other examples include the *apsRS* in *Staphylococcus epidermidis* and *graRS* in *S. aureus* that, in addition to transporting AMPs away from the site of action, are also capable of upregulating the *dlt* operon and *mprF* to increase the bacterial cell surface charge [49].

The sensitive-to-antimicrobial-peptides (Sap) uptake ABC transporter has been implicated in conferring tolerance to LL-37 [51,52,53,54]. The SapA is believed to bind to and transport LL-37 from the periplasm to the cytoplasm for degradation, all while avoiding direct contact between LL-37 and the cytoplasmic membrane [51]. During this process, the SapBC channels use the energy provided by the SapDF to facilitate the uptake of LL-37 into the cytoplasm [52]. Mutations in the SapBC resulted in higher susceptibility to LL-37 than mutations in the SapA, indicating that the SapBC retains partial permease activity in the absence of the SapA substrate binding, and thus therapeutic agents should focus on targeting the SapBC activity [52]. The mutations in the Sap permease complex increase vulnerability to LL-37 as there is higher accumulation of the peptide in the periplasmic space [12,54]. In addition, as seen in a study by Mount et al., the Sap transporter does not confer resistance to all bacteria containing the *SapABCDF* operon, or at least not all to the same extent. Mutations in the SapA of *Haemophilus influenzae* led to an 8-fold reduction in survival, while *Haemophilus influenzae* saw a 25% reduction [51].

The resistance-nodulation-cell division (RND) family of efflux pumps has also been implicated in LL-37 resistance, including the multiple transferable resistance (MTR) transporters in *H. ducreyi* [53], *Neisseria meningitidis* [55,56], and *Neisseria gonorrhoeae* [55]. Furthermore, the MefE transporter from the major facilitator superfamily (MSF) family protects *S. pneumoniae* against macrolides antibiotics, but also provides additional protection against LL-37 [32,57].

#### 2.1.3. Incorporation of Exogenous Molecules into the Bacterial Membrane

Another membrane modification that has been shown to increase bacterial resistance to LL-37 is the incorporation of exogenous cholesterol into the inner bacterial membrane. As described by McGee et al., *H. pylori* cells grown in the presence of cholesterol were over 100-fold more resistant to LL-37 than the control cells. The authors found that *H. pylori* is capable of stealing the cholesterol from the surrounding environment, modifying it through glycosylation, and incorporating it into the cell surface via cholesterol glucosyltranserase. The cholesterol glucosyltransferase is encoded by the *cgt* gene. While *cgt* mutants retained the cholesterol-dependent resistance to LL-37, there was an increased vulnerability to killing by colistin, which suggests that the pathogenesis is linked to the lipid A structure. While it is not yet fully understood, it appears likely that the cholesterol increases bacterial resistance by inducing modifications to the surface properties, such as an increased hydrophobicity, membrane rigidity and LPS structure alterations, and a decreased affinity to positively charged antimicrobials [58]. Rho family GTPases, which are involved in the host cell signalling, are abundantly present in cholesterol-rich lipid rafts, and thus bacteria can exploit these systems to increase their adhesion-induced resistance [56]. Nystatin, a lipid-raft inhibitor sequesters the cholesterol and restores LL-37 binding to the bacteria [56]. A study by Goytia and Shafer report that polyamines are another molecule that can be imported into the bacterial membrane. Extracellular polyamines can be incorporated into the membrane to interfere with LL-37, as well as other antimicrobials that target the cytosol and/or periplasm [59].

#### 2.1.4. Outer Membrane Proteins and Vesicles

OMVs and OMPs are utilized by bacteria to enhance resistance to LL-37 [60]. OMVs employ multiple methods to improve survival, including nutrient acquisition, biofilm development, removal of misfolded proteins, absorption of AMPs [61], and it has been suggested that OMVs transport virulence factors into the host cells [60]. OMVs are continuously being discharged from Gram-negative surfaces, where they can entrap phospholipids, LPS, periplasmic components [60], peptidoglycan, ion metabolites, nucleic acid, signalling molecules and OMPs [62]. OMPs play a role in the biogenesis of OMVs, as well as in the stabilization of the bacterial outer membrane [60,62]. As described by Lin et al., OmpA of *Acinetobacter baumannii* (abOmpA) is a trimeric porin that is important in virulence and transport of solutes, and it is theorized to bind to LL-37. When LL-37 binds to AbOmpA, it can inhibit motility and adhesion of *A. baumannii*; although *A. baumannii* is considered a non-motile bacterium, it has the capability to migrate under certain conditions [63]. In *Escherichia coli* and other enterobacteria, OmpA acts as an adhesion and invasion [63], and outer membrane protein A-like proteins (OmpALPs) in *Porphyromonas gingivalis* serves as a protection against LL-37 accumulation on the surface of the cell [64]. Mutations in both the *ompA* [63] and OmpALP [64] results in a greater sensitivity to LL-37. It is suggested that in the absence of *ompA*, LL-37 will bind to other OMPs that exhibit higher permeability and pore-forming ability, which increases the level of cell death [63]. In the absence of OmpALPs, LL-37 can accumulate on the cell surface, leading to enhanced destruction of the cell surface [64]. Moreover, Urashima et al. report that *ompT* expression augments the production of OmpT-loaded OMVs, contributing to LL-37 tolerance. OmpT-associated LL-37 resistance is more prominent in Enterohemorrhagic *E. coli* (EHEC) than in Enteropathogenic *E. coli* (EPEC) due to differences in the *ompT* gene expression [61]. The higher levels of OmpT proteins in EHEC encourage the degradation and inactivation of alpha-helical AMPs to promote survival, while the levels in EPEC had a minimal role in AMP degradation [65]. EHEC can cleave LL-37 at dibasic sequences, leading to proteolytic fragments that lack all antimicrobial activity [65], whereas the low OmpT levels in UPEC also does not contribute as significantly to LL-37 resistance [25]. That being said, if UPEC strains with high protease activity have the ompT-like gene *arlC* along with the *ompT* gene, there is an associated fitness advantage [66]. The *arlC* has also been implicated in resistance to CAMPs in the Adherent-invasive *E. coli* (AIEC) phenotype [67]. Furthermore, Chang et al. state that in Enterobacteriaceae, the Lpp outer membrane lipoprotein is proposed to form a channel through the outer membrane of the bacteria in order to block antimicrobial agents. Mutants lacking Lpp exhibit blebbing, periplasmic enzyme leakage, and issues with septa formation during cell division [68]. In *P. aeruginosa*, the outer membrane protein I plays a large role in the susceptibility to LL-37 [69].

#### 2.1.5. Proteases

Proteolytic cleavage and degradation is another common mechanism used to enhance resistance to antimicrobials, including LL-37 [20,21]. Proteases can be within the cell membrane, they can act intracellularly, or they can be secreted to function as exotoxins [70]. In addition, bacteria can recruit host cell proteases to promote the invasive spread of pathogens [32]. For instance, the streptokinase virulence factor recruits host plasminogen, activates plasminogen to plasmin, and binds plasmin to the surface of the bacteria where it will function to disrupt tissue barriers and degrade LL-37 [32,71]. In addition, metalloproteases have been implicated in LL-37 tolerance in several bacteria, including *P. aeruginosa* [72], *Bacillus anthracis* [29], and *Tannerella forsythia* [73]. While metalloproteases assist in the pathogenesis of several bacteria, metalloprotease inhibitors can be effective in blocking the harmful effects on LL-37, such as the EDTA, 1,10-phenanthroline [29] and LasB inhibitors [72]. Other proteases implicated in LL-37 resistance include the ClpXP intracellular protease [74], the SpeB [32] and SspB secreted cysteine proteases [75], and the PASP [64], aureolysin and V8 secreted proteases [75].

#### 2.1.6. Regulation of Gene Expression, Virulence and Phenotypic Changes

In response to incoming threats, bacteria have the ability to upregulate virulence encoding genes. In the presence of LL-37, bacteria can increase the expression of several toxic metabolites and enzymes [76], including SpeB, streptococcal inhibitor of complement (SIC), streptokinase, M1 [28], secretory proteins [27], and exopolysaccharides [9], which assist in the evasion of LL-37.

A study by Strempel et al. looked at the ability of LL-37 to promote the production of virulence factors and adaptive resistance in *P. aeruginosa*. This study concluded that exposure to LL-37 resulted in the upregulation of genes that contribute to the production of quorum sensing molecules, virulence factors, multidrug efflux pumps, in addition to inducing modifications to LPS. Moreover, *P. aeruginosa* cells that were previously treated with LL-37 showed an upregulation in the *mexGHI-opmD* operon and *pqsABCDE* genes. The upregulation in the *mexGHI-opmD* operon leads to alterations in the regulation of N-Acyl homoserine lactone (AHL) and Pseudomonas quinolone signal (PQS) molecules, both of which are involved in quorum sensing and virulence of *P. aeruginosa*. Quorum sensing molecules are important in cell-to-cell communication of *P. aeruginosa*, as well as swarming motility, formation of biofilms, iron binding, susceptibility to antibiotics, and virulence. The upregulation of the *pqsABCDE* genes also altered PQS signalling, as seen by the increased levels in the bacterial supernatant. [76].

As part of the adaptive stress response of *S. pyogenes*, the CovRS (control of virulence regulatory sensor kinase) will induce a transition from the colonizing phenotype to the invasive phenotype [77]. The CovRS, also known as the CsrRS, is a TCS that governs 15% of the expression of the *S. pyogenes* genome [28]. The invasive phenotype is strongly correlated with virulent infections seen in animal models; it mimics what is seen naturally in human CovRS mutant infections, and results in resistance to opsonophagocytic killing in in vitro human blood [77]. The LL-37-induced invasive phenotype includes the upregulation of virulence factors, the promotion of signal transduction, and the formation of vesicle-like structures on the cell surface that contribute to bacterial resistance [28]. The upregulated virulence factors include streptodornase D (*sdzD*) [78], streptolysin O (*slo*) [77,78,79], streptokinase (SK) [78], IL-8 protease (*spyCEP*) [77,78], hyaluronic acid capsule [77,79], DNase (*sda1*) [77], mac-IgG protease (*mac/IdeS*), NAD-glycohydrolase (*nga*), and pyrogenic exotoxin A (*speA*) [78]. In contrast, Tran-Winkler found that the invasive phenotype downregulates a cysteine protease (*speB*) and G-related α_2_-macroglobulin-binding protein (grab). The repression of *speB* contributes to virulence as the cysteine protease has been suggested to degrade the M1 anti-phagocytic protein, as well as inactivate *sda1*, which normally works to degrade NETs [78]. SpeB inactivation results in the ability for SK, M1 protein, and host plasminogen to persist at the site of infection, therefore allowing for the accumulation of plasmin on the bacterial surface, and the subsequent systemic spread of the infection [80]. The grab protein amplifies the activity of SpeB; therefore, the downregulation of *grab* further contributes to the antimicrobial resistance [32]. In contrast to CovRS switching, Mairpady Shambat et al. identified that a naturally occurring single amino acid substitution in the AgrC of methicillin-resistance *S. aureus* (MRSA) can destabilize the AgrC-AgrA, converting the phenotype from cytotoxic to colonizing. The colonizing phenotype is less susceptible to LL-37, yet causes less severe damage to the skin tissue [81].

A spontaneous mutation in the CovRS regulon can result in the M1T1 invasive serotype, which promotes resistance to neutrophils through the upregulation of Sda1 virulence factor [80]. This hypervirulent serotype seen in *S. pyogenes* also provides additional protection against LL-37 through the neutralization of the peptide and some of its derivatives [82]. LaRock et al. state that the M1T1 mutants lacking the M1 protein were more vulnerable to LL-37 killing. It appears that the increased sensitivity to LL-37 is not due to the cell alterations resulting from the protein deletion, but the direct ability of the M1 protein to sequester LL-37 [82]. The M1 protein does not solely contribute to the enhanced resistance of M1T1 serotypes, but also the Sda1 and the hyaluronic acid capsule [80].

Anionic exopolysaccharide capsules are located on the outer envelope of bacterial cells and serve as a virulence factor for many pathogens. The capsule can increase tolerance to LL-37 in multiple ways, including masking charge modifications and providing a sink [12]. *S. pyogenes* mutants lacking a hyaluronic acid capsule are more vulnerable to killing by LL-37, and other capsule mutations sensitized *Streptococcus iniae* and *S. pneumoniae* to LL-37 [32].

Another factor affecting the expression of virulence factors is the variable morphology of bacteria. For instance, the isogenic type II *Burkholderia pseudomallei* has small, rough colonies and an increase in the formation of biofilm and production of proteases, whereas the isogenic type II *B. pseudomallei* has large, smooth colonies and an increase in the expression of flagella [83].

#### 2.1.7. Metabolic Changes

Metabolic changes are another mechanism by which bacteria gain resistance. In order for positively charged molecules such as LL-37 to be imported into the bacteria, an electrochemical gradient is required. Therefore, reductions in the electron transport have been reported to provide stable AMP resistance to *S. aureus* [19]. In contrast, mutants with complete disruption in the electron transport chain (ETC) and defects in the biosynthesis and metabolic pathways are more sensitive to LL-37 in comparison to the wild type *S. aureus* strain [33]. In addition, Wuersching et al. suggest that oxygen availability alters the antimicrobial activity of LL-37. Facultative anaerobic bacteria *S. mutans*, *Streptococcus sanguinis*, and *Actinomyces naeslundii* were less susceptible to LL-37 in comparison to the obligate anaerobic bacteria *Veillonella parvula*, *Parvimonas micra*, and *Fusobacterium nucleatum* [84]. These results are supported by a study by Eini et al., which found that *E. coli* also has a decreased resistance to LL-37 under anaerobiosis. That being said, the effects of oxygen are likely dependent on the strain of bacteria, as *S. pyogenes* aerotolerant bacteria has an increased resistance to LL-37 under oxygen deprivation [85].

### 2.2. Cross-Resistance

Cross-resistance poses an additional challenge when attempting to make LL-37 into a viable treatment option. There are current reports of cross-resistance between LL-37 and colistin [86,87,88], and polymyxin B [27,89]. There is risk of further resistance developing with other antimicrobial agents that target bacteria using similar modes of action as LL-37 [90]. Moreover, a review by Fleitas and Franco presents an additional concern that using AMPs, such as LL-37, as therapeutic agents will lead to cross-resistance with AMP constituents of the human immune response, thus jeopardizing the human’s natural defense against pathogenic species [91].

#### 2.2.1. Colistin

Colistin, also known as polymyxin E, is a CAMP. It is a last resort treatment option for multidrug-resistant, Gram-negative bacterial infections [92]. Colistin targets bacteria using similar mechanisms to LL-37; colistin binds to the lipid A portion of LPS, which causes disruptions to the membrane integrity, and leads to cell death [86]. The similar modes of action have led to speculation of cross-resistance. Several studies have found cross-resistance between colistin and LL-37 [86,87,88], yet other studies show no correlation [93,94]. A positive correlation has been found in *A.* baumannii clinical isolates [88], as well as strains of *K. pneumoniae* [87]. As seen in a study by Jayol et al., mutations in certain genes, such as the *pmrB* and *mgrB* genes, were also found to confer cross-resistance. The *pmrB* and *mgrB* genes are involved in the PmrAB and PhoPQ TCS systems, respectively, meaning both pathways are implicated in LPS biosynthesis. The *pmrB* mutation leads to constitutive activation of PmrA. The activation of PmrA leads to the upregulation of three loci, all of which cause the LPS to be more positively charged, and thus decreases the affinity of CAMPs to the bacterial membrane [92]. In addition, Al-Farsi et al. report that *mgrB* insertions are linked to cross-resistance between colistin and LL-37. The *mgrB* encodes a negative feedback regulator, which results in the upregulation of the Pmr lipopolysaccharide modification system. This study found that *K. pneumoniae* isolates with *mgrB* insertions display cross-resistance, but only when the concentration of the LL-37 is above 50 μg/mL. This suggests that the issue of cross-resistance may be most prevalent when LL-37 is present in high concentrations, such as during an infection or inflammation [86]. In contrast with the previously described study [86], García-Quintanilla and colleagues found that the complete loss of LPS expression in *A. baumannii* could alter the susceptibility to colistin, but has insignificant effects on the antimicrobial activity of LL-37. This finding suggests that the mechanisms of colistin and LL-37 may differ more than what has been initially proposed [93]. In addition, mutations in the *mcr-1* phosphoethanolamine transferase neither conferred cross-resistance in *E. coli*, nor in *K. pneumoniae;* this is not the expected result, considering the MCR-1 masks the negatively charged phosphates in the LPS, thus promoting tolerance [94]. Therefore, the results determining whether or not bacteria can develop cross-resistance between LL-37 and colistin is largely inconclusive [86].

#### 2.2.2. Polymyxin B

Polymyxin B (PmB) is a cationic polypeptide that is used against Gram-negative pathogens. PmB belongs to the same class of drug as colistin, and thus their mechanisms of action are similar. LPS is the initial target; once the LPS is destabilized through electrostatic interactions, the permeability of the membrane is increased, causing cell lysis and death [95]. Therefore, since the mechanisms of action are similar, PmB holds a potential risk for cross-resistance with LL-37. A study from Duperthuy et al. looked at how OMVs contribute to the development of cross-resistance with AMPs. The study concluded that when OMVs from *Vibrio cholerae* were grown in sub-lethal concentrations of PmB, they were later less susceptible to LL-37. It was found that exposure to PmB increased the size of the OMV being released by *V. cholerae.* In addition, the vesicles contained a biofilm-associated extracellular matrix protein (Bap1), which is suggested to act as a scaffolding protein that reduces the amount of free LL-37 that is capable of killing bacteria [27]. A study by Tzeng et al. found cross-resistance in *N. meningitidis* with mutations in the type IV pilin biogenesis, supporting the notion that PmB exposure can lead to tolerance against LL-37 [89].

#### 2.2.3. Chlorhexidine

Chlorhexidine is a CAMP that is frequently used as an antiseptic in health care settings. To kill bacteria, chlorhexidine targets membrane fluidity, which results in the loss of structural integrity of the membrane [96]. It has been reported that cross-resistance can develop between chlorhexidine and colistin [96], which causes concerns that a similar tolerance can develop against LL-37. A study by Hashemi et al. has determined that resistance of Gram-negative bacteria to chlorhexidine led to an increased tolerance to colistin, yet the bacteria remains susceptible to LL-37 [97].

### 2.3. Mutations

Mutations in bacteria not only occur naturally as a result of errors in DNA replication, but it has also been reported that LL-37 can act as a mutagen [98,99]. Rodríguez-Rojas et al. highlight that LL-37 can promote mutagenesis through the facilitation of free iron into bacteria. Once inside the bacterial cell, free iron can interact with hydrogen peroxide which can lead to the formation of reactive oxygen species, and subsequent DNA damage. This form of mutagenesis has been witnessed with *Pseudomonas aeruginosa*, as cystic fibrosis infections results in elevated levels of free iron in the body [98]. Limoli and Wozniak describe how LL-37 can also induce mutagenesis through the promotion of mucoid conversion. Once inside the bacterial cell, LL-37 can disrupt the DNA replication process, and induce mutations that allow for the overproduction of a protective polysaccharide alginate coating [99]. Specifically, LL-37 can disrupt MucA, a negative regulator of alginate synthesis, which promotes transcription factors within the alginate biosynthesis operon to overproduce alginate [99,100,101].

Mutations or deletions of genes in certain bacteria may alter the cell properties such that the vulnerability of the bacteria to LL-37 is enhanced. Among such cell property changes include modifications to the charge, hydrophobicity and permeability of the bacteria, as well as changes to the virulence factors, metabolism and overall structure of the bacterial cell envelope (Table 1). Specifically, the disruption to virulence factors includes a reduced production and/or expression of staphylokinase [55,102], hyaluronic acid capsule [80], LPS [26,40,44,103], curli fibers [104], pustules [51,52], outer membrane vesicles (OMV) [61,65], antimicrobial peptide detoxification modules and other efflux pump transporters [49,105], alginate [100,101], pili [106], alkyl hydroperoxide reductase [49], and alcohol dehydrogenase [49]. The disruption in biofilm formation refers to either a decrease in the attachment [33] or adhesion [63]. In some cases, multiple genes have the same effect on a bacterium because the expression of one gene may be dependent on the expression or presence of another gene, or one gene may be a regulator of the target gene; therefore, a mutation in one gene has an effect on both genes. For example, *dltC* is regulated by CiaRH in *Streptococcus mutans* biofilm cells [37], the expression of *lrgAB* is dependent on the presence of ClpX [74], and *zapB* is needed for the recruitment and activity of EnvC [107]. In some cases, the precise effect of the gene is unknown, although there are suggested mechanisms based on the known role of the gene in the bacteria [30,64].

### 2.4. Biofilms

Biofilms are aggregates of bacteria that are associated with at least 65% of all microbial infections, and 80% of chronic infections [110]. In comparison to their planktonic counterparts, biofilm cells are better protected from antimicrobial agents [111]. Among the reasons for the enhanced tolerance of biofilms cells include slow growth rates and presence of persister cells within the biofilms [7,112], as well as limited antimicrobial activities due to nutrient starvation, and poor penetration and diffusion into the biofilms [113]. Similar to planktonic cells, biofilm cells have a number of resistance mechanisms that facilitate the evasion from LL-37.

Curli are surface-associated amyloid fibers in bacteria that enhance resistance by binding to LL-37 before the peptide has the chance to contact the bacterial cell membrane [114]. In biofilm formation, curli fibers assist in the adhesion to surfaces and the aggregation of cells [104,114]. Curli fibers have been reported to enhance the resistance of uropathogenic *E. coli* (UPEC) biofilms to LL-37 [104,114]. Curli and cellulose are both highly expressed in *E. coli* clinical isolates and have complementary effects on the bacteria [114], where bacteria expressing the curli-positive, cellulose-negative phenotype display enhanced virulence [104]. MS7163, a curli-producing UPEC strain has a mutation in the *bcsA* gene responsible for cellulose production, and therefore has a heightened tolerance to LL-37 [104]. However, at low concentrations, LL-37 is capable of polymerizing CsgA, the major curli subunit, and therefore inhibits curli formation and prevents in vitro curli-mediated biofilm formation [114].

In addition, small colony variants (SCV) are a subpopulation of cells that exhibit a slow growth rate, and thus have a high capacity to form biofilms [115]. The rugose small colony variant (RSCV) phenotype provides protection against LL-37 in bacteria including *S. aureus* [116] and *P. aeruginosa* [117]. Pestrak et al. state that this phenotype involves mutations that result in the excessive production of c-di-GMP. These mutations result in hyper-biofilm forming strains that overproduce the exopolysaccharides Pel and Psl, which facilitates the formation of bacterial aggregates. Both polysaccharides play an important role in the initial attachment and formation of biofilms, and the resistance to antimicrobial agents [117].

Notably, the vulnerability of planktonic cells to a particular antimicrobial agent is not indicative of the vulnerability of the biofilm cells [118,119,120]. For instance, LL-37 has shown to display strong biofilm prevention against strains of *S. aureus* that are resistant to LL-37 [118]. Likewise, in high salt environments, LL-37 loses its antimicrobial activity, yet retains its anti-biofilm activity [121]. LL-37 is being studied for its anti-biofilm activities as it has been reported to be effective in targeting bacteria at different stages of biofilm growth and development; LL-37 has not only been shown to prevent biofilm formation in various bacteria including *S. aureus* and *P. aeruginosa* [8,121,122,123], but also inhibits the attachment of cells [121,124,125], and disrupts pre-formed and mature biofilms [122,124,126,127,128]. According to a study conducted by Kang et al., LL-37 has superior anti-biofilm activity when compared to both silver nanoparticles and conventional antibiotics [20].

A review by Iacob and Iacob has listed the characteristics of LL-37, which has enabled it to be effective in the killing of biofilm cells. These characteristics include its small size, positive net charge, large antibacterial spectrum, LPS-neutralizing effects, low resistance rates, synergistic effects, and immunomodulation [7]. The exact mechanism by which LL-37 kills biofilm cells is not fully understood, although it is probable that LL-37 is able to penetrate the biofilm and exert its bactericidal effects against the embedded bacteria [20]. Other suggested mechanisms include the induction of the bacterial SOS response, the promotion of twitching motility, and the disruption of intracellular quorum-sensing molecules [8]. Ideally, LL-37 would be used as a preventative measure as opposed to being a treatment against pre-formed biofilms, as they are more difficult to combat [129,130].

## 3. Methods to Improve LL-37 as a Therapeutic Agent

### 3.1. Immobilization Techniques and LL-37 Delivery Systems

Peptide immobilization techniques provide a means to overcome some of the challenges associated with LL-37, including cytotoxicity and low stability in physiological environments [131,132,133,134]. Immobilization may increase the ability of LL-37 to inhibit the colonization and biofilm formation of bacteria [131,132,134], and thus immobilization techniques are utilized in wound environments [131] and medical devices [132]. Likewise, delivery systems allow for the encapsulation and controlled release of LL-37, which improves cytotoxicity, proteolytic degradation, and the poor penetration of LL-37 in deep wounds [135,136,137,138,139,140]. Delivery systems have been shown to be an effective method by which the stability and antimicrobial activity can be enhanced. However, this review will not describe delivery systems in depth, as recent reviews have previously covered this information in great detail [141,142].

### 3.2. LL-37 Derivatives

The aim of deriving new antimicrobial drugs from LL-37 is to retain the beneficial qualities of the original peptide, while removing the undesirable qualities. According to Nagant et al., three main drawbacks of LL-37 should be taken into account when developing a new drug: LL-37 is a long peptide and it is costly to synthesize, LL-37 is vulnerable to proteases, and LL-37 exhibits toxicity and hemolysis against eukaryotic cells [143]. There are many methods by which LL-37 derivatives can be made: The amino acid sequence can be altered, truncated, combined with another antimicrobial agent to make a hybrid, or a combination of these methods can be used (Table 2). Table 2 depicts LL-37 derivatives that have been reported as having superior antimicrobial qualities in comparison to the parent peptide. The improved qualities may be related to several characteristics of LL-37, including but not limited to the cell selectively [144,145,146], hemolysis and cytotoxicity [144,147,148], stability [124,135], biofilm prevention and disruption [33,143,149,150], and wound healing properties [146,151]. While these derivatives may demonstrate improvements over LL-37 in certain facets, several articles have reported shortcomings of particular derived peptides, of which include inferior binding abilities [144], limited antibacterial spectrum [121,147], weaker membrane disruptions [147], reduced activities in certain biological fluids [135], higher hemolysis and cytotoxic effects [145,148,152], and reduced capabilities against biofilm cells [153,154]. In this section, we will focus on the properties of LL-37—the sequence, helicity, hydrophobicity, charge, and configuration—and how alterations to these properties can change the antimicrobial activity of LL-37.

#### 3.2.1. Sequence

Different regions of the LL-37 amino acid sequence have been implicated in certain roles and functions of the peptide. When deriving a new drug from LL-37, it is important to consider how adding or removing certain regions and/or amino acids will change the antimicrobial properties of LL-37. In general, LL-37 derivatives tend to be shorter in length than the parent peptide (Table 2). Not only is this important in reducing the cost of synthesis, but also for improving the antimicrobial activity, as shorter sequences have been found to more easily penetrate the bacterial cell membrane and the biofilm matrix [146].

The N-terminal sequence of LL-37 comprises residues 1–12 [175]. This region of LL-37 begins with two leucine amino acids, which are associated with a high degree of peptide instability [152]. The N-terminal region is responsible for resistance to proteases, oligomerization, chemotaxis, hemolysis, and peptide aggregation [8]. It has been shown that the N-terminal only has antimicrobial activities against pathogens that are highly susceptible to the peptide, indicating that this region does not play a significant role in the killing of bacteria [176]. In fact, removal of the N-terminal has been shown to enhance the bactericidal activities of LL-37 [146,157,176,177,178]. A study by Kanthawong et al. described how removing the first six residues reduces the bacterial activities of LL-37, while further removal by 12 and 18 residues mostly restores the activity [157]. This is supported by another study which found that LL-20 is nearly inactive, while LL-32 has increased activity compared to LL-37 [8]. In addition to truncations, Kamysz et al. have observed the effects of modifications to the N-terminal sequence. The antimicrobial activity of KR12-NH2, a shorter analog of LL-37, was examined after modification to the N-terminus. The conjugation of fatty acid chains to the N-terminus was seen to enhance activities against highly drug-resistant ESKAPE pathogens (*Enterococcus faecium*, *S. aureus*, *K. pneumoniae*, *A. baumannii*, *P. aeruginosa*, and *Enterobacter* spp.), as well as *S. aureus* biofilms. While the antimicrobial activities of the peptide were improved, a higher cytotoxicity was examined against human red blood cells and the HaCaT keratinocyte cell line [178]. In the design of LL-37-derived peptides, many opt for the complete removal of the N-terminal sequence, as removing these residues has also been shown to reduce the cytotoxicity [20] and the hemolytic properties of LL-37 [146], while not compromising the stability of the peptides [146].

The C-terminal LL-37 helix has been shown to be responsible for the antimicrobial activities of LL-37 [8], while the linear terminal tail residues 32–37 do not play a role in the peptide’s interactions with bacterial membranes [152]. Multiple studies have indicated that the truncation of residues 31–37 will not impact the bactericidal activities of LL-37, although further truncation does reduce the killing abilities of the peptide [112,152,157]. This corresponds with studies that conclude that the main antimicrobial and anti-biofilm properties of LL-37 resides in the central helix, specifically in residues ~17–31 [132,146,149]. The smallest fragment that retains the antimicrobial activities of LL-37 is KR-12, which corresponds to residues 18–29 of the parent peptide [131,154]. In addition, residues 26–31 were demonstrated to be particularly important in antimicrobial potency, and derivatives lacking this sequence—LL-13, LL-19, LL-25, RK-19, and IG-13—had little to no bactericidal activities [112]. Similar to the N-terminus, the C-terminal tail region is involved in peptide aggregation, and thus the removal of the last six to seven residues has been shown to increase the overall bactericidal potency of LL-37 [176].

The composition of amino acids is a critical determinant for the antibacterial activity spectrum. This is evident as certain LL-37 derivatives, such as KR-12 and GF-17d3, are mostly active against Gram-negatives pathogens, whereas 17BIPHE2-3RA is more active against Gram-positive staphylococcal species [179]. Moreover, Wang et al. found that peptides that are effective at killing Gram-negative bacteria are more likely to have a higher net charge and a lower hydrophobic content than peptides that are active against Gram-positive bacteria. Consequently, basic amino appear to be important in the ability of LL-37 to kill Gram-negative bacteria, while they have little impact on the antimicrobial activity against Gram-positive bacteria. The authors demonstrated this trend by observing the changes in MIC values after lysines were substituted for alanines. The MIC of Gram-negative *A. baumannii* increased, while the values of Gram-positive *S. aureus* USA300 and S. *epidermidis* had no significant change [179]. Another study by Jahangiri et al. highlights the importance of basic residues, particularly Phe17, Ile24, Phe27 and Val32, in the hydrophobic interactions that facilitate deep penetration into the cell membrane. In addition, Asp26 creates repulsive forces with the membrane that creates unfavourable interactions with the bacteria [149]. A single amino acid substitution of aspartic acid for valine at this position was able to confer antimicrobial activities in VQ-12, which is an inactive fragment of LL-37 [146]. Phenylalanine is further emphasized as an important amino acid in LL-37, as it directly interacts with the anionic phosphatidylglycerol in bacterial membranes [173]. That being said, tryptophan-containing AMPs exhibit more potent antimicrobial activities in comparison to phenylalanine- or tyrosine-containing peptides. This is possibly because tryptophan increases the net positive charge and hydrophobicity, which may lead to improved interactions with the bacterial membrane [145,154]. In addition, tryptophan at position 27 reduced the hemolysis of 17tF-W, an LL-37-derived peptide [173]. Furthermore, Arg23 and Lys25 were critical for the binding of GF-17 to both Gram-negative and Gram-positive model membranes, as these residues are directly exposed to the membrane [149]. This corresponds to the findings of other studies, which found that Arg23 in GF-17 and 17BIPHE2 has an important role in permeating the membrane and killing the bacteria [21]. Arginine, when substituted in place of glutamic acid, creates a Lys-Arg motive that has been reported enhance the antibacterial activities of LL-37-derived peptides [165].

#### 3.2.2. Helicity

The alpha helical content of LL-37 is approximately 34% in phosphate buffer saline (PBS) solution [180]. The helicity of LL-37 increases upon binding to the bacterial membrane, which is a common characteristic of lipid-binding peptides [180]. Helical conformation is associated with the binding to Gram-negative membranes [179], and deep penetration into the cell membrane of bacteria [163]. Higher helicity has been shown to improve hemolytic activity, as this structure promotes selectivity for the anionic membranes of bacteria over the zwitterionic membranes of mammalian cells [164]. An increase in the alpha helical structure has also been correlated with a greater antibacterial potency in numerous studies. LL-37 derivatives with a higher helical content than the parent peptide, including, M-L [162], LL-32 [156], KR-12-a5 [161], AL32-P113, and L31-P113 [152], had stronger antimicrobial activities. However, high helicity is not always indicative of improved bactericidal activities; IG-19 was equally effective as LL-37 in killing *Burkholderia* species despite having half of the propensity to adopt alpha helices, whereas LL-25 has a similar propensity to LL-37 yet had roughly a third of the killing abilities [112]. Additionally, Ulaeto et al. state that at physiological salt concentrations, the alpha helical conformation of LL-37 is enhanced, while its bactericidal activity is inhibited. Therefore, it was concluded in one study that homologues of LL-37 that had a lower propensity to adopt this conformation had more effective bactericidal activities. The authors also declare that the adoption of alpha helical structure plays a role in antimicrobial activities, as does the time at which this conformation is assumed. Preformed helices have greater difficulties disrupting bacterial membranes, and optimal bactericidal activity may depend on adopting the helical conformation prior to interactions with the target membrane [181]. Overall, it appears that helical content is not an adequate predictor of antibacterial activities [146,166].

#### 3.2.3. Hydrophobicity

The hydrophobicity of LL-37 plays a role in membrane disruptions and bactericidal activities. Following the adsorption of LL-37 onto the bacterial cell membrane, the hydrophobic residues are able to facilitate further penetration into the membrane, and to disorganize the lipid tail region of the membrane [120]. In addition, amphiphilicity—the separation of charged groups from hydrophobic residues—promotes interactions with the membrane and penetration into the lipid bilayer [153]. The typical amphipathic, alpha helical structure has hydrophobic residues and positively charged residues distributed on each side of the peptide. BMAP-27 adopts this particular structure and has a stronger antimicrobial activity compared to LL-37, where these residues are more dispersed throughout the sequence [120]. KR12 has superior amphipathic helices compared to LL-37 [153], and the analogs with the highest hydrophobicity were better able to inhibit LPS-stimulated tumor necrosis factor-α production, as well as exhibit higher LPS-binding activity [154]. However, higher hydrophobicity has been correlated with an increased cytotoxicity [160] and worse cell selectively in LL-37 [145]. Multiple studies have indicated that lowering the hydrophobicity leads to a reduction in cytotoxicity [10,160], and a decrease in cell selectively against mammalian cells [145,149]. Modifications to the sequence of LL-37, such as a leucine to alanine substitution, are able to reduce the hydrophobicity of the peptide and promote these benefits [152]. A study by Tan et al. contrasts the previous findings, indicating that the ideal amphiphilicity is achieved when the positively charged residues and the hydrophobic residues are more dispersed. This study contradicts other studies, indicating that an increased hydrophobicity is correlated with lower hemolysis and improved cell selectively [164]. It has been suggested that there is an optimal hydrophobicity window, where raising or lowering the hydrophobicity outside of this range drastically decreases antimicrobial activity [182].

#### 3.2.4. Charge

Net charge of the peptide also plays a role in antimicrobial activities and should be considered in the design of novel peptides. There is a net negative charge in both Gram-negative and Gram-positive bacteria, from the LPS and lipoteichoic acids, respectively. Several studies have indicated that derivatives with an increased cationicity are better able to kill the bacteria, as stronger electrostatic interactions lead to a higher affinity for the bacterial membrane [120,152,163,164]. Conversely, analogs of an LL-37-derived peptide that have a lower net charge of +4 and +5 showed increased bactericidal activities compared to the parent peptide [178]. Likewise, Aghazadeh et al. demonstrated that some peptides with a significantly higher cationic charge, such as P22 (+10), exhibited worse bactericidal activities compared to the parent. Perhaps, the positive charge density on the peptide exceeds a critical threshold, and thus compromises the antimicrobial activity. Another consideration is that there needs to be a balance between the charge and hydrophobicity of the peptide. In addition to a high charge, P22 has a low number of hydrophobic residues, which may contribute to its low bactericidal activities [166]. A study by Jacob et al. found that antimicrobial activity is not dependent on charge, although a certain ratio between hydrophobicity and charge of the peptides was found to increase the antimicrobial activity, as well as LPS neutralization effects [154]. The balance may need to be carefully considered, as peptides with a high cationicity and hydrophobicity exhibited a greater affinity for the zwitterionic membranes of human red blood cells, causing larger hemolytic activities [152]. A study by Caiaffa et al. suggests that absolute positive charge is not correlated with antimicrobial activity, yet there is an association with the percentage of positive charge in the peptide [161]. Overall, the correlation between charge and antimicrobial activities is complex, and it is suggested that alpha helicity and amphiphilicity may play a more important role in the design of peptides [164].

#### 3.2.5. Configuration

The configurational stereochemistry of a peptide can alter its antimicrobial effects. LL-37 is naturally in an L-configuration, although the D-enantiomer also displays antimicrobial activities. Studies have looked at the change in bactericidal effects after the incorporation of d-amino acids, and they found that the activities are relatively similar [124,127]. In fact, some have witnessed improved antibacterial abilities compared to L-LL-37 [121,157,171]. D-LL-37 may promote more wound healing effects, as it has been reported to induce IL-8 in keratinocytes and stimulate the proliferation of fibroblasts [121]. Furthermore, many studies have reported that D-LL-37 is also able to effectively combat biofilm infections. The killing abilities of D-peptides against *Burkholderia thailandensis* [127], *S. aureus* [121], and *P. aeruginosa* [124] biofilms were comparable to L-LL-37, while certain studies have found that D-peptides exhibited improved activities against *B. pseudomallei* [171], *Mycobacterium avium* [183], and *P. aeruginosa* [184] biofilms. Similar to LL-37, D-LL-37 is capable of inhibiting the attachment of bacteria, disrupting pre-formed biofilms, and promoting twitching motility by downregulating the expression of certain genes, such as *rhlA* and *rhlB* [124]. In addition, multiple studies report that the D-configuration of peptides may be more resistant to proteolytic degradation than the L-configuration. This is seen as D-LL-37 is resistant to degradation from trypsin, whereas L-LL-37 is not resistant, and thus experiences a loss of function [121,124,127]. The incorporation of d-amino acids into the peptide sequence results in a similar hemolytic activity [171], yet these amino acids were also attributed with a significant improvement in cell selectively [160]. While most studies observed benefits in using the D-configuration, one study concluded that the EC50 value of the D-peptide was higher for *S. aureus* than LL-37. This decrease in potency may be due to a reduced ability of the D-enantiomer to interact with the Gram-positive membrane, or perhaps it is due from lower helicity compared to the L-enantiomer [121]. Moreover, an additional study concluded that LL-37 enantiomers exhibited negligible activity compared to *de-novo* designed alpha helical AMPs [182].

### 3.3. Synergy

There are several reasons for using more than one antimicrobial agent against a pathogenic species, such as targeting drug-resistant bacteria and reducing the level of toxicity towards human cells [185]. Zharkova et al. state that in many cases, synergistic pairs were effective even when the bacteria showed a moderate to high level of resistance against one of the antimicrobial substances. Therefore, if a bacterium develops resistance against a particular antimicrobial agent, finding synergism is a method to allow for the continued effective treatment of that bacterium. In addition, using multiple antimicrobial substances reduces the quantity of each drug needed to kill the bacteria, potentially reducing the toxicity against eukaryotic cells [185]. Nevertheless, there are several challenges associated with the co-formulation of antimicrobial agents; Chauhan et al. outline the current analytical, technical, regulatory, and economical barriers, including adverse interactions between the drugs, and lower product shelf life than the monotherapies. Despite the challenges, there are in fact several co-formulated drugs that are currently available for clinical use, such as co-amoxiclav, a combination of amoxicillin and clavulanic acid. Chauhan et al. state that the coformulation of antimicrobial agents is a useful tool so long as the clinical benefits outweigh the associated obstacles [186].

In this section, we will focus on the synergistic combinations that enhance LL-37’s ability to kill bacteria, including those that are effective at killing and preventing biofilms. Synergism may result from combining LL-37 with another antimicrobial agent, or two different antimicrobial agents can synergistically combine to indirectly enhance the antibacterial potency of LL-37. A large portion of the literature describes synergy between LL-37 and antibiotics, which has been summarized in Table 3. It is important to note that the strains of bacteria that were susceptible to these combinations were included in this table, as the synergistic effects are highly strain specific. The same combination may show either indifferent, additive, or antagonistic effects against a different strain of the same bacterium. While combinations with antibiotics comprise a great quantity of the research, combinations with other antimicrobial agents are also described in this review.

#### 3.3.1. Combinations with LL-37

Synergy is commonly found in combinations with AMPs that target the bacterial membrane and antibiotics that target the biosynthesis of nucleic acids and proteins [185]. AMPs, in this case, LL-37, would increase the permeability of the bacterial membrane and facilitate the entry of the antibiotics into the cell where they can exert their antimicrobial effects [177,185,195,198]. Furthermore, many studies have proposed that the ability of an antimicrobial agent to synergize with LL-37 depends on whether it is a bactericidal or a bacteriostatic substance. It has been suggested that synergism results when bactericidal agents are paired together, or when bacteriostatic agents are paired; the combination of bacteriostatic and bactericidal substances will lead to an antagonistic interaction [177,191]. Leszczyńska et al. also suggest that the synergistic effects between LL-37 and the bactericidal substance are enhanced if the antibiotic targets the bacterial wall structure. A possible mechanism to explain the antagonistic effect between LL-37 and bacteriostatic antibiotics is that the bacteriostatic substance triggers the bacterial stress response. Bacteriostatic antibiotics may lead to an increase in the expression of multidrug resistance efflux pumps, which will expel LL-37 into the extracellular environment, and will therefore decreases its susceptibility to the peptide [177]. Conversely, a study by Nuding et al. suggests that the synergistic effects with LL-37 is not dependent on whether the other antimicrobial agent is bacteriostatic or bactericidal. The same study also indicated that the ability of LL-37 to synergize with antibiotics is not dependent on the mode of action of the antibiotic either [195]. LL-37 has shown synergistic effects with antimicrobial agents that target the cell wall, the nucleic acid synthesis, and the protein synthesis of bacteria.

##### Cell Wall Inhibitors

LL-37 produces synergistic effects upon pairing with antimicrobial agents that target the cell membrane of bacteria, including beta-lactams [199], peptide antibiotics [13,197], glycopeptides [38,150], and ceragenins [113]. LL-37 synergizes with beta lactams, such as nafcillin [199] and meropenem [195], as well as beta lactam/beta lactamase inhibitor (BLI) combinations, including piperacillin/tazobactam [195]. The exact mechanism for the enhanced activity is unknown, although it appears to do less with protecting beta lactams from beta-lactamase enzymes, and more to do with the how the compounds bind to penicillin-binding proteins (PBPs) in order to disrupt the cell wall synthesis and metabolism of bacteria [190]. Avibactam, a non–beta-lactam BLI, interacts with the penicillin-binding protein 2, which interferes with the cell wall dynamics of multidrug-resistant *K. pneumoniae,* thus sensitizing the bacteria to subsequent LL-37 exposure [196]. In addition, LL-37 is able to synergize with peptide antibiotics, including colistin [197] and SMAP-29 [13]. Colistin, paired with LL-37, was shown to potentiate the ability of azithromycin to kill Stenotrophomonas maltophilia by increasing the drug entry, facilitating access to its ribosomal target, and potentially sensitizing the bacteria to neutrophils [197]. A study by Chung et al. reported that when LL-37 is paired with the sheep cathelicidin SMAP-29, there is an increased binding of LL-37 to AcpP, which is a cofactor for bacterial fatty acid biosynthesis. This cofactor plays an important role in bacterial growth, and the overexpression of this gene leads to an increased susceptibility of *Francisella novicida* to LL-37. LL-37-AcpP binding may be a broad mechanism of action, as LL-37 has shown to bind to the AcpP in *E. coli* and *B. anthracis* as well [13]. Moreover, LL-37 has shown synergism with several glycopeptides, including teicoplanin [38] and vancomycin [150]. It has been proposed that teicoplanin binds to, and masks the D-alanyl residues to increase the affinity of LL-37 to the bacterial membrane [38]. Vancomycin can synergize with AMPs that disrupt cell walls, including LL-37; the synergy likely results from the increased drug exposure at the division septum of the cell, where vancomycin exerts its antimicrobial activity [150]. As discussed by Wnorowska et al., one problem of current commonly used antimicrobial agents is that they are effective at eradicating extracellular pathogens, yet they display poor retention when inside of the host cells. The synergistic pair of LL-37 with ceragenins were effective at eradicating intracellular *E. coli*, most likely because both compounds use electrostatic forces to bind to the bacterial membrane in order to cause bacterial cell death [113].

##### Nucleic Acid Synthesis Inhibitors

LL-37 has shown the potential to synergize with antimicrobial agents that target the bacterial nucleic acid synthesis, such as moxifloxacin [195]. That being said, Zharkova et al. reported that AMPs are more likely to synergize with inhibitors of protein synthesis than with inhibitors of nucleic acid synthesis. It has been suggested that the inhibitors of nucleic acid synthesis act against the bacteria at slower rates in comparison to inhibitors of protein synthesis. Another potential reason for the decrease in synergy is that nucleic acid inhibitors, including ofloxacin and rifampicin, have rigid structure of fused aromatic rings that restrict the ability to penetrate through the pores that the AMP form in the bacterial membrane [185].

##### Protein Synthesis Inhibitors

LL-37 is reported to have synergistic effects when paired with antibiotics that affect protein synthesis of bacteria, including tigecycline [195], gentamicin [185], and azithromycin [188]. The combination of LL-37 and azithromycin was shown to increase the permeability of multidrug resistant Gram-negative rods; the pair may also initiate a positive feedback loop that increases the active intracellular levels of azithromycin [188]. While many of the reported synergistic combinations include LL-37 paired with an antibiotic, LL-37 has also been shown to synergize with other antimicrobial agents that target the protein synthesis of bacteria. Fumakia and Ho found that when LL-37 is combined with serpin A1, an elastase inhibitor, the LPS is neutralized and the permeability of the epithelial cell barrier is increased. Serpin A1 primarily enhances the antimicrobial activities of LL-37 through the protection against endotoxins, and aiding in the suppression of bacterial proliferation [138]. KKL-40, a small-molecule inhibitor of trans-translation, causes a maladapted stress response in the bacteria to LL-37, thus increasing the bacteria’s vulnerability to the peptide [200].

##### Other Combinations

Other synergistic combinations include LL-37 with nanoparticles and bacteriocins. When paired with gold-coated nanoparticles, there was an increased antimicrobial activity against *P. aeruginosa* and *S. aureus* [201]. Likewise, it appears that LL-37 facilitates the entry of gold nanoparticles (AuNPs) into the cell, resulting in an increased angiogenesis and improved wound healing [202]. When pairing LL-37 with double hydroxide nanoparticles, it is important to consider the size of the particle; smaller sized particles are more capable of binding to, and disrupting the anionic membrane of bacteria [203]. Furthermore, as described by Bitschar et al., LL-37 can synergize with the lanthionine-containing bacteriocins (lantibiotics) that are produced by commensal bacteria on the human skin. This shows that bacterial AMPs can potentially act in synergy with human AMPs to defend against pathogenic bacteria [204]. The commensal coagulase-negative staphylococci, *Staphylococcus hominis* [205], *Staphylococcus simulans* [139], and *Staphylococcus lugdunensis* [204] have shown to synergize with LL-37 to protect against epithelial colonization of pathogenic species, including *S. aureus*. Lugdunin, which is a peptide isolated from *S. lugdunensis*, was reported to promote the expression and release of LL-37 in keratinocytes [204]. The mechanism of lysostaphin, a metallo-endopeptidase produced by *S. simulans*, is unknown, although it is likely that it causes cell wall degradation and makes the cell membrane more accessible to LL-37 [139].

#### 3.3.2. Synergy with LL-37 Derivatives

As described above, numerous LL-37-derived peptides have been reported to be more effective in the killing of pathogenic bacteria. Likewise, LL-37 derivatives have been reported to work in synergy with several antibiotics to more efficiently kill bacteria. A summary of these results can be seen in Table 4, where both the LL-37 derivatives and antibiotics that comprise the synergistic pair are listed, as well as the genus and strain of bacteria that were found to be vulnerable to this combination.

#### 3.3.3. Synergy against Biofilms

LL-37 is capable of synergizing with several antimicrobial agents, including ceragenins [113], ciprofloxacin [126], and nanoparticles [201,206] in order to more effectively prevent and eradicate biofilms. For instance, the combination of LL-37 and ciprofloxacin decreased the minimum biofilm eradicating concentration (MBEC) values up to eight-fold [126]. A study conducted by Mishra and Wang found that antibiotics and peptides alone could disperse biofilms when the growth time was less than eight hours, and all peptides tested are able to combat initial attachment and biofilm formation. However, all peptides except for 17BIPHE2 required combinations with antibiotics in order to kill mature biofilms [134]. A study by Mishra et al. supports these findings, as they reported 17BIPHE2 and GF-17 could disrupt attachment, growth and established biofilms, while LL-37 could neither inhibit attachment nor disrupt pre-formed biofilms [168]. Other combinations involving LL-37-derived peptides, including D-LL-31 [171,172], IG-13-1, IG-13-2 [159], SAAP-148, and SAAP-276 [38] show enhancements in the anti-biofilm activities compared to the individual responses.

#### 3.3.4. Combinations That Enhance LL-37

The combination of daptomycin (DAP) and ceftaroline (CPT) have been reported to potentiate the antimicrobial activities of LL-37. CPT is a broad-spectrum, cephalosporin antibiotic that binds to PBPs in order to inhibit the cell wall synthesis of bacteria [207], and DAP is a lipopeptide that targets bacteria through the disruption of cell membrane integrity [208]. DAP exhibits antibacterial effects against heterogeneous VAN-intermediate *S. aureus (hVISA)*, *VISA* [208], MRSA and vancomycin-resistant enterococci (VRE) [207]. CPT displays activity against hVISA, VISA, VAN-resistant *S. aureus* (VRSA) and DAP-nonsusceptible (DNS) *S. aureus* [207]. One study found that the combination of DAP and CPT increased the amount of LL-37 binding to the surface of VRE, and therefore sensitizes the bacteria to LL-37 [209]. The combination has also been shown to sensitize *Enterococcus faecalis* to LL-37 [210]. Another study examined the combination of DAP and CPT against 26 staphylococcal bacteremia, which included 20 MRSA cases. This synergistic combination was reported to enhance the ability of LL-37 to kill the MRSA bacteria [211]. It has been proposed that the enhanced activity of LL-37 that results from the combination of DAP and CPT is mediated by an increase in negativity of the bacterial membrane, leading to stronger electrostatic interactions [207]. In addition, ampicillin is a penicillin antibiotic that targets the cell wall synthesis of bacteria. Combination of ampicillin with DAP also displayed synergistic effects, which led to an enhanced ability of LL-37 to kill AMP- and VAN- resistant *E. faecium* clinical isolates [193].

## 4. Final Remarks

It has previously been assumed that AMPs would be less prone to bacterial resistance in comparison to conventional antibiotics. Indeed, LL-37 has multiple bactericidal targets and may be less likely to induce resistance, although the full extent of AMP bacterial tolerance is largely unknown. Recent research has confirmed the induction of resistance following the use of sub-inhibitory concentrations of LL-37, as well as ability of LL-37 to function as a mutagen. Several mechanisms of resistance have been proven to be effective in the evasion of LL-37 defenses, including charge modifications, cell envelope alterations, efflux transporters, proteases, and metabolic changes. In response to LL-37, bacteria can also upregulate the expression of multiple virulence factors and can undergo phenotypic changes to become more invasive. Moreover, there is enhanced resistance seen in biofilm cell populations, and the potential for cross-resistance to develop between LL-37 and other antimicrobial agents. Additionally, since AMPs are a component of the innate immune system, there is added concern that the use of synthetic AMPs in a clinical setting will promote bacterial tolerance to effectors of the human innate immune response. If this is the case, the human’s natural defenses against invading pathogens would be compromised [91].

In addition to the challenges associated with bacterial resistance, there are several undesirable properties of LL-37, such as toxicity to mammalian cells and the inability to retain antimicrobial activity in physiological environments. The implementations of immobilization techniques and delivery systems, the development of LL-37 derivatives, and the discovery of synergistic combinations may eliminate the undesirable effects, while also improving the antibacterial and anti-biofilm properties of the peptide. Alterations to LL-37, including changes to the amino acid sequence, charge, helicity, hydrophobicity, configuration and synergistic pair are among the strategies that have been identified to improve LL-37’s antimicrobial activities. The modifications to the structure and the method of administration may also allow for LL-37 to better combat resistant strains of bacteria.

There are many factors currently hindering the success of LL-37 as a novel therapeutic agent. Future research may focus on understanding the extent to which bacteria can become resistance to LL-37, and how to enhance its antimicrobial and anti-biofilm capabilities. Once there is clear insight regarding the resistance development against LL-37, the strategies listed above can be exploited to create a peptide capable of circumventing such bacterial resistance. Several strategies have proven to be effective in isolation, although a combination of these methods may prove to be the most successful in optimizing LL-37 in the treatment of bacterial infections.

## Figures and Tables

**Table 1:** Mutations or deletions in genes that result in an increased susceptibility to LL-37 killing.

Bacteria	Gene	Effect of Mutation/Deletion of Gene	Reference
*Pseudomonas* *aeruginosa*	*vacJ*	Increased membrane permeability	[108]
	*algD*	Disruption to virulence factors	[100,101]
*Neisseria* *gonorrhoeae*	*mpg*	Disruption to virulence factors	[106]
	*pilE*	Disruption to virulence factors	[106]
*Streptococcus* *mutans*	*dltC*, *ciaRh*	Disruption in biofilm formation	[37]
*Streptococcus* *agalactiae*	*dltA*	Decreased cell surface charge	[36]
	*bceR*	Disruption to virulence factors	[49]
*Staphylococcus* *aureus*	*pheS*, *mprF*, *graR*, *vraF*, *trkA*	Increased membrane permeability	[33]
	*lspA*, *vraR*	Disruption in biofilm formation	[33]
	*argC*, *lipA*, *pheS*, *yrF*, *thrB*, *acoB*	Disruption to biosynthesis and metabolism	[33]
	*secDF*; *blaI*	Disruption to virulence factors	[56,102]
	*dltA*, mprF	Decreased cell surface charge	[38]
*Streptococcus* *pneumoniae*	*dltD*, *licD2*; *dlt*	Decreased cell surface charge	[12,35]
*Acinetobacter* *baumannii*	*ompA*	Disruption in biofilm formation	[63]
*Helicobacter* *pylori*	*lpxE*, *lpxF*	Disruption to virulence factors, and decreased cell surface charge	[40]
*Bacillus anthracis*	*clpX*, *lrgAB*	Structural changes to cell envelope	[74]
*Clostridioides* *difficile*	*clnR*, *clnAB*	Disruption to biosynthesis and metabolism, and disruption to virulence factors	[105]
*Mycobacterium* *tuberculosis*	*rv1258c*	Increased membrane permeability	[109]
*Salmonella enterica*	*phoPQ*	Disruption to virulence factors	[26]
	*envC*, *zapB*	Decreased cell surface charge, increased hydrophobicity	[107]
*Escherichia coli*	*yrfF*, *rcpA*; *ompT*, *pch*, *ler*, *lrp*; *ompT*	Disruption to virulence factors	[61,65,104]
	*envC*, *zapB*	Decreased cell surface charge, increased hydrophobicity	[107]
	*waaP*	Disruption to virulence factors	[44]
*Enterococcus faecalis*	*liaR*	Structural changes to cell envelope	[30]
*Porphyromonas* *gingivalis*	*pgm6/pgm7*	Structural changes to cell envelope	[64]
*Streptococcus* *pyogenes*	*gacI*	Increased hydrophobicity	[39]
	*hasA*	Disruption to virulence factors	[80]
*Haemophilus ducreyi*	*cpxA*, *mtrC;* *sapBC*, *sapA*	Disruption to virulence factors	[51,52,53]
*Haemophilus* *influenzae*	*sapBC*, *h*trB**	Disruption to virulence factors	[54]
*Vibrio cholerae*	*msbB*	Disruption to virulence factors	[103]

**Table 2:** LL-37 derivatives that display enhanced antimicrobial activity when compared to the LL-37 parent peptide.

Type of Derivative	LL-37 Derivative	Sequence	Reference
WT sequence	LL-37	LLGDFFRKSKEKIGKEFKRIVQRIKDFLRNLVPRTES	[155]
Truncations (without amino acid substitutions)	LL-32	LLGDFFRKSKEKIGKEFKRIVQRIKDFLRNLV	[156]
	LL-31 *	LLGDFFRKSKEKIGKEFKRIVQRIKDFLRNL	[112,143,157]
	IG-19	LLGDFFRKSKEKIGKEFKR	[157]
	RK-31	RKSKEKIGKEFKRIVQRIKDFLRNLVPRTES	[143]
	RK-25	RKSKEKIGKEFKRIVQRIKDFLRNL	[143]
	LL7-27	RKSKEKIGKEFKRIVQRIKDF	[147]
	KS-30 *	KSKEKIGKEFKRIVQRIKDFLRNLVPRTES	[158]
	LL-13 *	IGKEFKRIVQRIKDFLRNLVPRTES	[150]
	KE-18 *	KEFKRIVQRIKDFLRNLV	[153]
	LL-17 *	FKRIVQRIKDFLR	[150]
	FK-12	FKRIVQRIKDFL	[146]
	KR-20 *	KRIVQRIKDFLRNLVPRTES	[158]
	KR-12 *	KRIVQRIKDFLR	
Truncations (with amino acid substitutions)	IG-13-1	IGK**L**FKRIVQ**L**IK	[159]
	IG-13-2	IGK**L**FKRIVQ**L**I**L**	[159]
	FK13-a1 *	**W**KRIV**R**RIK**RW**LR	[145]
	FK13-a7 *	**W**KR**W**V**R**R**W**K**RW**LR	[145]
	KR-12-a2	KRIVQRIK**KW**LR	[154]
	KR-12-a3	KRIV**K**RIK**KW**LR	[154]
	KR-12-a4	KRIV**KL**IK**KW**LR	[154]
	KR-12-a5 *	KRIV**KL**I**LKW**LR	[154,160,161]
	KR-12-a6	**L**RIV**KL**I**LKW**LR	[154]
	VQ-12^V26^	VQRIK**V**FLRNLV	[146]
Hybrids (with truncated LL-37)	AL32-P113	A**LGDFFRKSKEKIGKEFKRIVQRIKDFLRNL**AKRHHGYKRK FHLEY	[152]
	L31-P113	**LGDFFRKSKEKIGKEFKRIVQRIKDFLRNL**AK	[152]
	M-L	GIGAVLKVLTTGL**FKRIVQRIKDFLRN**	[162]
	B1	KFKKLFKKLSPV**FKRIVQRIKDFLR**	[148]
	C-L	KWKLFKKI**FKRIVQRIKDFLRN**	[163]
	FV-LL *	FRIRVRV**FKRIVQRIKDFLR**	[164]
	LI	**GKEFKRIV**KWPWWPWRR	[144]
Peptides based on LL-37	LLAP	GRKSAKKIGKRAKRI	[165]
	P38	TSVRQRWRWRQRVRTS	[166]
	RP557 *	RFCWKVCYKGICFKKCK	[167]
	GF-17 *	GFKRIVQRIKDFLRNLV	[149,168]
	OP-145 *	IGKEFKRIVERIKRFLRELVRPLR	[116,169]
	P60.4Ac *	IGKEFKRIVERIKRFLRELVRPLR	[151]
	P10 *	LAREYKKIVEKLKRWLRQVLRTLR	[151]
	SAAP-145	LKRLYKRLAKLIKRLYRYLKKPVR	[135]
	SAAP-148 *	LKRVWKRVFKLLKRYWRQLKKPVR	[135,170]
	SAAP-159	LKRLYKRVFRLLKRYYRQLRRPVR	[135]
	SAAP-276	LKRVWKAVFKLLKRYWRQLKKPVR	[135]
D-enantiomers	D-LL-31 *	LLGDFFRKSKEKIGKEFKRIVQRIKDFLRNL	[171,172]
	D-LL-37	LLGDFFRKSKEKIGKEFKRIVQRIKDFLRNLVPRTES	[121,124]
	17tF-W *	GX_1_KR*l*VQR*l*KDW*l*RKLV, where *1* is a D amino acid and X1 = 4-*t*-butylphenylalanine	[173]
	17BIPHE2 *	GX_1_KR*1*VQR*1*KDX_2_*1*RNLV, where *1* is a D amino acid and X_1_ = X_2_ = biphenylalanine	[33,134,168,173,174]

WT wild-type. * LL-37-derived peptide with anti-biofilm activity in addition to its antibacterial properties. Substituted amino acids in the truncated peptides are represented in bold text. In the hybrid peptides, the amino acid sequence belonging to the LL-37 parent peptide is represented in bold text.

**Table 3:** Combinations of LL-37 and antibiotics that show synergistic effects against specific pathogens.

Bacteria	Antibiotic Paired with LL-37	Isolate	Reference
*Pseudomonas* *aeruginosa*	Colistin	AS1, MDRPA1, MDRPA2; 2 CIs	[126,187]
	Imipenem	MDRPA1	[187]
	Azithromycin	PA01	[188]
	Ciprofloxacin	2 CIs	[126]
	Tobramycin	1 CI	[189]
*Staphylococcus* *aureus*	Tazobactam	Sanger 252, VISA D712, hVISA D592	[190]
	Tobramycin	4 CIs	[189]
	Teicoplanin	JAR060131, ATCC 49230, AMC201, LUH15101	[38]
	Vancomycin	ATCC 25923	[150]
	Amoxicillin with clavulanic acid	ATCC 29213, 3 MSSA CIs, 2 MRSA CIs	[177]
	Amikacin	MSSA CI	[177]
	Nafcillin	MSSA CIs	[191]
*Enterococcus* *faecium*	Ceftaroline	DAP-susceptible parent strain, R6370, 8019	[192]
	Ampicillin	DAP-susceptible parent strain, 8019; AMP- and VAN-resistant isolate	[192,193]
	Ertapenem	DAP-susceptible parent strain, R6370, 8019	[192]
	Oritavancin	VAN-resistant CI	[194]
	Oritavancin + Ampicillin		
*Enterococcus* *faecalis*	Ceftaroline	R6981	[192]
	Ertapenem	R6981	[192]
*Clostridioides* *difficile*	Moxifloxacin	9 toxinogenic and 10 non-toxinogenic CIs, DSM 1296	[195]
	Tigecycline		
	Piperacillin-tazobactam		
	Meropenem		
*Klebsiella* *pneumonaie*	Azithromycin	K700603	[188]
	Avibactam	CDC1100192, KP1088 and KP1004	[196]
	Zidebactam	CDC1100192	[196]
*Acinetobacter* *baumannii*	Tazobactam	AB5075, AB1 AB2, AB3, AB4	[190]
	Azithromycin	Ab19606	[188]
*Stenotrophomonas maltophilia*	Colistin	K279a (ATCC BAA-2423)	[197]
*Micrococcus luteus*	Gentamicin	CIP A270	[185]
*Streptococcus spp.*	Tobramycin	2 CIs	[189]

AS, antibiotic susceptible; MDRPA, multi-drug resistant *Pseudomonas aeruginosa*; CI, clinical isolate; MSSA, methicillin-susceptible *Staphylococcus aureus*; VISA, vancomycin intermediate *Staphylococcus aureus*; DAP, daptomycin; VAN, vancomycin.

**Table 4:** Combinations of LL-37-derived peptides and antibiotics that show synergistic effects against specific pathogens.

Bacteria	Antibiotic	LL-37 Derivative	Strain	Reference
*Pseudomonas* *aeruginosa*	Ciprofloxacin	KR-12-a5, KR-12-a5(6-^D^L), KR-12-a5(7-^D^L)	MDRPA (CCARM 2095)	[160]
	Rifampicin	B1	ATCC BAA-2114	[148]
	Oxacillin	KR-12-a5, KR-12-a5(5-^D^K), KR-12-a5(7-^D^L)	MDRPA (CCARM 2095)	[160]
	Chloramphenicol	FK13; KR-12-a5, KR-12-a5(5-^D^K), KR-12-a5(6-^D^L)	MDRPA (CCARM 2095)	[145,160]
*Staphylococcus* *aureus*	Ampicillin	B1	BAA-41	[148]
	Levofloxacin	B1	ATCC 33591, ATCC BAA-41	[148]
	Chloramphenicol	B1; FK13-a1, FK13-a7; C-L	ATCC 43300; MRSA (CCARM 3095); ATCC 25923	[145,148,163]
	Erythromycin	B1	ATCC 33591	[148]
	Thiamphenicol	C-L	ATCC 25923	[163]
	Neomycin sulfate	C-L	ATCC 25923	[163]
	Rifampicin	B1	ATCC 33591, BAA-41	[148]
*Burkholderia* *pseudomallei*	Ceftazidime	D-LL-31	1026b, H777, M10	[171]
*Escherichia coli*	Neomycin sulfate	C-L	ATCC 25923	[163]

MDRPA1 multi-drug resistant *Pseudomonas aeruginosa*.
